# Short-term increase in the carriage of azithromycin-resistant *Escherichia coli* and *Klebsiella pneumoniae* in mothers and their newborns following intra-partum azithromycin: a post hoc analysis of a double-blind randomized trial

**DOI:** 10.1093/jacamr/dlaa128

**Published:** 2021-01-27

**Authors:** Pauline Getanda, Abdoulie Bojang, Bully Camara, Isatou Jagne-Cox, Effua Usuf, Benjamin P Howden, Umberto D’Alessandro, Christian Bottomley, Anna Roca

**Affiliations:** 1 Disease Control and Elimination Theme, Medical Research Council Unit The Gambia at London School of Hygiene and Tropical Medicine, Fajara, The Gambia; 2 Microbiological Diagnostic Unit Public Health Laboratory, Department of Microbiology and Immunology, University of Melbourne, Doherty Institute for Infection and Immunity, Victoria, Australia; 3 Faculty of Infectious and Tropical Diseases, London School of Hygiene and Tropical Medicine, London, UK

## Abstract

**Objectives:**

To evaluate the impact of one oral dose of intrapartum azithromycin (2 g) on the carriage and antibiotic resistance of *Escherichia coli* and *Klebsiella pneumoniae* in the nasopharynx, breast milk and vaginal swabs of mothers and *K. pneumoniae* in the nasopharynx of their newborns.

**Methods:**

We performed a post hoc analysis of a double-blind, placebo-controlled randomized-trial (ratio 1:1) conducted in The Gambia. Breast milk (BM) and vaginal swabs (VS) from mothers and nasopharyngeal swabs (NPS) from mother–newborn pairs were collected at different timepoints during the 4 week follow-up. Samples were processed using standard microbiological procedures. For BM and NPS post-intervention results were combined for analysis.

**Results:**

In the original trial 829 mothers were randomized. In this analysis, complete sample sets were available for 630 mothers for *E. coli* analysis (76.0%) and 564 mother–newborn pairs for *K. pneumoniae* analysis (68.0%). For *E. coli*, carriage prevalence in BM and VS was similar in both arms but resistance was higher in the azithromycin arm in VS (2.6% versus 0%, *P = *0.004). For *K. pneumoniae*, carriage prevalence was higher in the azithromycin arm for BM (13.8% versus 8.7%, *P = *0.055) but not for VS or NPS. Prevalence of azithromycin resistant *K. pneumoniae* was higher in the azithromycin arm for BM (3.6% versus 1.0%, *P = *0.050) and VS (1.5% versus 0% *P = *0.057).

**Conclusions:**

Oral intrapartum azithromycin did not reduce carriage of *E. coli* and *K. pneumoniae* and was associated with an increase in the prevalence of azithromycin-resistant *E. coli* and *K. pneumoniae* isolates in BM and VS.

## Introduction

Despite substantial efforts to reduce worldwide neonatal mortality, it remains unacceptably high, with up to 2.5 million deaths per year. The greatest mortality burden falls on low- and middle-income countries, with countries in West and Central Africa, and South Asia having the highest rates in 2017.^1^ Infections, mostly sepsis and meningitis, account for one-third of these deaths.^2^

Both Gram-positive and Gram-negative bacteria are responsible for neonatal sepsis in sub-Saharan Africa, with recent data pointing to *Staphylococcus aureus* as the most common causative agent, closely followed by Enterobacterales, i.e. *Escherichia coli* and *Klebsiella* spp.^3^ Antimicrobial resistance (AMR) in *S. aureus* is relatively low on the African continent, but AMR particularly to ampicillin and gentamicin (antibiotics recommended by WHO for the treatment of neonatal sepsis) is much higher for *E. coli* and *Klebsiella* spp.^3^ The most clinically significant *Klebsiella* species is *Klebsiella pneumoniae* because of its higher prevalence. Furthermore, its role together with *E. coli*, in hospital- and community-acquired infections is alarming,^3–5^ as they may harbour genes that confer resistance to β-lactam antibiotics, fluoroquinolones and aminoglycosides that are carried on transferable mobile genetic elements.^6^ Infections with MDR Enterobacterales are particularly difficult to treat and are associated with poor outcomes.^7^

Context-specific interventions aimed at reducing the high burden of neonatal sepsis are urgently needed in sub-Saharan Africa. We conducted a double-blind randomized trial (PregnAnZI) to assess the effect of one oral dose of intrapartum azithromycin (2 g) on bacterial carriage of the main Gram-positive pathogens associated with neonatal sepsis [*S. aureus*, *Streptococcus pneumoniae* and Group B *Streptococcus,* (GBS)] in the mother and the newborn during the 4 weeks following treatment.^8^ The trial showed a reduction of bacterial carriage of these three bacteria in both the mothers and their newborns during the entire neonatal period and lower occurrence of clinical disease in treated mothers (i.e. mastitis, sepsis and fever) and their newborns (i.e. skin sepsis and overall infections) during the 8 weeks following the intervention.^9^

In order to further assess the effect of intrapartum azithromycin on the risk of sepsis, here, we evaluate its effect on the prevalence of carriage and antibiotic resistance of *E. coli* and *K. pneumoniae* in mothers and *K. pneumoniae* in their newborns during the 4 weeks following the intervention.

## Patients and methods

### Study site

The study was conducted at the Bundung Maternal and Child Health Hospital [formerly known as Jammeh Foundation for Peace (JFP)], a government-run health centre located in western Gambia that manages 4500 deliveries each year. The population in the catchment area is representative of The Gambia and covers the main ethnic groups. Approximately 63% of deliveries in the country occur in health facilities.^10^ The climate of the area is typical of the sub-Sahel region, with a dry (January–June) and wet season (July–December). Illiteracy is high.^11^

### Trial design

The original trial was a double-blind, placebo-controlled randomized trial in which women attending the study health facility in labour were randomized to receive a single dose of oral azithromycin (2 g) or placebo (ratio 1:1).^12^ Women aged 18–45 years with no acute or chronic conditions were recruited between April 2013 and April 2014 when attending JFP labour ward. The women had provided written informed consent to participate in the study during previous antenatal care visits. Women and their newborns were followed for up to 8 weeks postpartum and biological samples were collected during the first 4 weeks.^8^^,^^12^ Further details of the trial are reported elsewhere. ^12^ Clinical Trials registration: NCT01800942.

### Trial samples

Pre-intervention samples [nasopharyngeal swab (NPS) and vaginal swab (VS)] were collected at the study health facility during labour and before the intervention was administered. An NPS was collected from the newborn within 6 h of birth. After discharge, NPS (mothers and babies) and breast milk samples were collected at days 3, 6, 14 and 28. In addition, a VS was collected between days 8 and 10 after delivery at the postnatal checkup visit that took place at the health facility. Details of sample collection can be found in the study protocol.^12^

An NPS was also collected at an additional visit (outside of trial follow-up period) for study infants at the age of 11–13 months.

### Ethics approval

A local safety monitor (LSM) and a data safety monitoring board (DSMB) reviewed serious adverse events during the trial, and the trial was monitored by an independent clinical trials monitor. The trial and the 12 months follow-up study were approved by the joint Gambia Government/Medical Research Council (MRC) Ethics committee.

### Trial status

The trial had been completed at the time of submission of this manuscript.

### Sample handling and laboratory methods

All the field and lab staff were blinded to the participant trial arm. NPS were collected using a calcium alginate swab that was inserted that was inserted to the posterior wall of the nasopharynx. The swab was rotated and left in the nasopharynx for approximately 5 s and then placed in a vial containing skim milk/tryptone/glucose/glycerol transport medium. The vial containing the inoculum was put into a cold box and transported to MRC laboratories within 8 h. Low VS were collected using a sterile cotton swab inserted 2–3 cm into the vagina. The swab was rotated and left in the vagina for approximately 5 s. The inoculated swabs were then immediately placed into vials containing skim milk/tryptone/glucose/glycerol, put in a cold box and transported to MRC laboratories within 8 h. For breast milk (BM) samples, the nipple and areola were disinfected using sterile cotton soaked with 0.02% chlorhexidine. Mothers were asked to manually express their milk, of which the first 0.5 mL was discarded and the following 1–2 mL collected in another sterile bijoux bottle. This were put in a cold box and transported to MRC laboratories within 8 h.^12^

On arrival in the laboratory, samples were vortexed for 20 s and stored at −70°C for batch processing. During processing, samples were first thawed on ice and then vortexed briefly to homogenize the medium. An aliquot of 50 μL was dispensed onto MacConkey agar (Oxoid, UK) and streaked for the selective isolation of *E. coli* and *K. pneumoniae.*

*E. coli* was isolated from mothers’ BM and VS while *K. pneumoniae* was isolated from these sample types in addition to mothers and newborns NPS.

#### Identification of E. coli and K. pneumoniae

After 20–24 h of incubation at 37°C, MacConkey plates were examined for growth of lactose-fermenting smooth pink colonies, presumptive for *E. coli*. Three to five suspected colonies were sub-cultured on blood agar and tested using Kovac’s indole test to differentiate *E. coli* from other lactose fermenters. Lactose-positive and indole-positive isolates were confirmed to be *E. coli*. Presence of mucoid, pink colonies was presumptive for *K. pneumoniae*. Three to five suspected colonies were sub-cultured on blood agar and subjected to biochemical testing using the rapid API 20E kit (bioMérieux, France) for the identification of *K. pneumoniae*.

#### Antibiotic susceptibility

Well-isolated colonies of *E. coli* or *K. pneumoniae* were transferred to 2.5 mL of sterile physiological saline to achieve turbidity of 0.5 MacFarland standard. A sterile swab that had been immersed into the suspension was streaked onto the surface of Mueller Hinton agar (Oxoid, UK) in three directions, rotating the plate at 90° angles to ensure even distribution. Azithromycin susceptibility was tested using disc diffusion (15 μg azithromycin disc) and the MICs of intermediate and resistant isolates were determined by Etest according to the CLSI guidelines.^13^ CLSI do not have clinical break points for azithromycin resistance in *E. coli* and *K. pneumoniae*, consequently isolates with MICs ≥32 mg/L were considered resistant based on epidemiological cutoff values for other Enterobacterales.

#### Statistical analysis

The data were double entered into Open Clinica and analysed using Stata 14. Only mothers from whom VS and BM samples were collected at all visits were included in the *E. coli* analysis. If in addition they and their newborns had NPS collected at all timepoints, the pair was included in the *K. pneumoniae* analysis. For each sample type, post-intervention carriage was considered if any of the post-intervention samples were positive. In addition, we also calculated the prevalence of carriage at each timepoint. Prevalence between arms was compared using prevalence ratios (PR) with their corresponding 95% CIs. We used the χ^2^ test to obtain *P* values for the prevalence of carriage and Fisher’s exact test for *P* values for the prevalence of resistance. For the post-intervention *K. pneumoniae* carriage analysis of the VS, we adjusted for the observed baseline imbalance using Poisson regression with robust standard errors. The statistical analysis was conducted using Stata version 13.1.

## Results

### Study population

**Figure 1.**
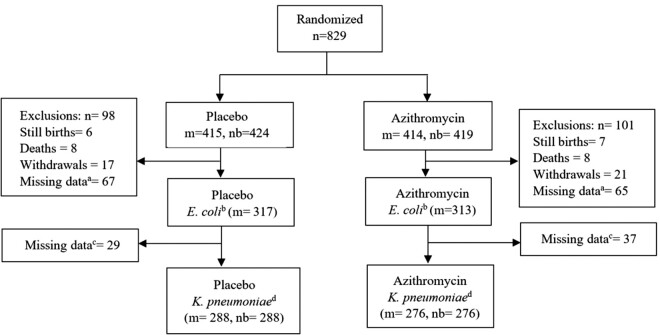
Study profile. Abbreviations: m, mothers, nb, newborns. Footnotes: ^a^Mothers with ≥1 missing sample; ^b^Samples from mothers; ^c^Mother*–*newborn pairs with ≥1 missing sample; ^d^Samples from mothers and newborns.

Eight hundred and twenty-nine women were recruited into the trial (*n = *414 in the azithromycin arm and *n = *415 in the placebo arm). These women delivered 843 newborns, including 13 stillbirths. Overall, 630 (76% of 829) and 564 (68% of 829) of mother–newborn pairs had all study samples collected and are part of this post hoc analysis for *E. coli* and *K. pneumoniae* respectively (Figure 1). Details of baseline characteristics of the participants included are shown in Table 1.

**Table 1.** Baseline characteristics of study participants

Characteristics	*K. pneumoniae*		*E. coli*	
	Azithromycin	Placebo	Azithromycin	Placebo
Mothers	*n = *276	*n = *288	*n = *313	*n = *317
Age, months, median (IQR)^a^	26.0 (22.0–30.0)	26.0 (22.0–30.0)	26.0 (22.0–30.0)	26.0 (22.0–29.0)
Ethnicity, *n* (%)				
Mandinka	104 (37.7)	126 (43.8)	119 (38)	143 (41.5)
Wollof	37 (13.4)	38 (13.2)	41 (13.1)	40 (12.6)
Jola	50 (18.1)	40 (13.9)	53 (16.9)	42 (13.2)
Fula	49 (17.8)	47 (16.3)	60 (19.2)	51 (16.1)
Other	34 (12.3)	37 (12.8)	38 (12.1)	41 (12.9)
Season of delivery^b^, *n* (%)				
Dry	182 (65.9)	200 (69.4)	203 (64.9)	215 (67.8)
Rainy	94 (34.1)	88 (30.6)	110 (35.1)	102 (32.2)
Mode of delivery, *n* (%)				
Vaginal	276 (100)	288 (100)	313 (100)	317 (100)
Multiple pregnancy, *n* (%)	2 (0.72)	4 (1.39)	3 (1.0)	7 (2.2)
Time from treatment to delivery, hours, median (IQR)	3.2 (1.1–8.3)	2.9 (1.3–6.3)	3.2 (1.1–8.3)	2.9 (1.3–6.3)
Time from rupture of membrane to delivery, hours, median (IQR)	0.4 (0.1–1.8)	0.3 (0.1–1.3)	0.4 (0.1–1.8)	0.3 (0.1–1.3)
Newborns^c^	*n = *276	*n = *288		
Gender, *n* (%)				
Females	138 (50)	132 (45.8)		
Males	138 (50)	156 (54.2)		
Gestational age, months, (IQR)	36.0 (34.0–38.0)	36.0 (35.0–38.0)		
Weight, kg, median (IQR)	3.1 (2.8–3.5)	3.1 (2.9–3.4)		

a Age missing in *n = *3.

b Dry season in The Gambia is November to May and Rainy season is June to October.

c Only *K. pneumoniae* was isolated from babies (NPS).

### Prevalence of E. coli carriage and resistance

#### Prevalence of carriage

**Figure 2.**
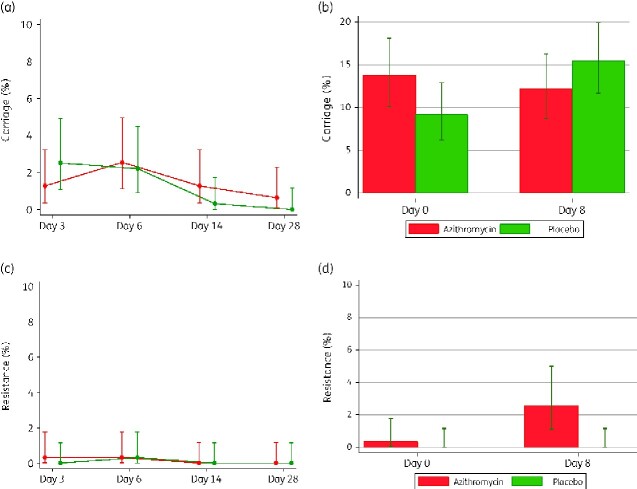
*E. coli* prevalence of carriage at different timepoints: (a) Breast milk carriage. (b) Vaginal carriage. (c) Breast milk resistance. (d) Vaginal resistance.

For the study women, prevalence of *E. coli* carriage between study arms was similar in both BM and VS. Overall post-intervention carriage was higher in VS compared with BM (15.5% versus 5.1%) (Table 2). Details of prevalence of carriage by trial arm at each visit are shown in Figure 2(a and b).

**Table 2.** Prevalence of *E. coli* carriage and resistance by study arm

	Azithromycin (%) (*n = *313)	Placebo (%) (*n = *317)	PR (95% CI)	*P* value
Prevalence of carriage				
Breast milk				
Post^a^	17 (5.4)	16 (5.1)	1.08 (0.55–2.09)	0.828
Vaginal swab				
Pre^b^	43 (13.7)	29 (9.2)	1.50 (0.96–2.34)	0.070
Post^c^	38 (12.1)	49 (15.5)	0.79 (0.53–1.16)	0.227
Prevalence of carriage of resistant isolates				
Breast milk				
Post^a^	2 (0.6)	1 (0.3)	2.03 (0.18–22.22)	0.622
Vaginal swab				
Pre^b^	1 (0.3)	0	–	0.497
Post^c^	8 (2.6)	0	–	0.004

PR, prevalence ratio. *P* values from χ^2^ test for prevalence of carriage and Fisher’s exact test for prevalence of resistance.

a Cumulative incidence of carriage/resistance, i.e. proportion positive at one or more post-treatment visits (days 3, 6, 14 and 28).

b Incidence of carriage/resistance at day 0.

c Incidence of carriage/resistance at day 8.

#### Prevalence of resistance

Prevalence of *E. coli* isolates resistant to azithromycin post-intervention was higher in the azithromycin arm only for the VS (2.6% versus 0% *P = *0.004) (Table 2). Details of prevalence of azithromycin-resistant isolates at each visit are shown in Figure 2(c and d).

### Prevalence of K. pneumoniaeK. pneumoniae carriage and resistance

#### Prevalence of carriage

For the study women, post-intervention prevalence of *K. pneumoniae* carriage was similar between arms for NPS (Table 3) but higher in the azithromycin arm for BM [13.8% versus 8.7% PR = 1.59, 95% CI 0.98–2.56) *P = *0.055] and VS (5.8% versus 1.4% PR = 4.17, 95% CI 1.41–12.33, *P = *0.004). However, pre-intervention prevalence of carriage in the VS was already higher in the azithromycin arm (4.0% versus 1.4% PR = 2.87, 95% CI 0.92–8.90, *P = *0.055) and after adjusting for this baseline imbalance, the difference was not significant (PR = 3.11, 95% CI 0.82–11.84, *P = *0.095). Details of prevalence of carriage by trial arm at each visit are shown in Figure 3(a-d).

**Figure 3.**
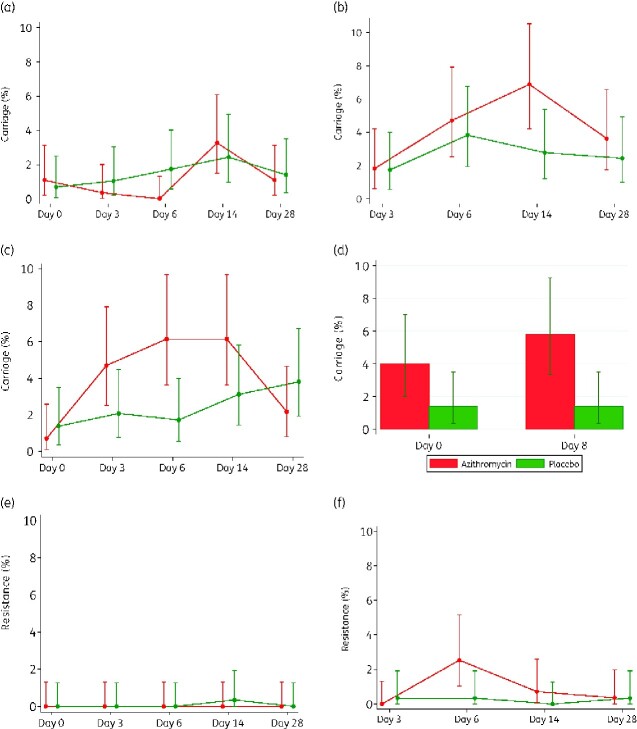
*K. pneumoniae* carriage at different timepoints. (a) Maternal NPS carriage. (b) Breast milk carriage. (c) Neonatal NPS carriage. (d) Vaginal carriage. (e) Maternal NPS resistance. (f) Breast milk resistance. (g) Neonatal NPS resistance. (h) Vaginal resistance.

**Figure 3.**
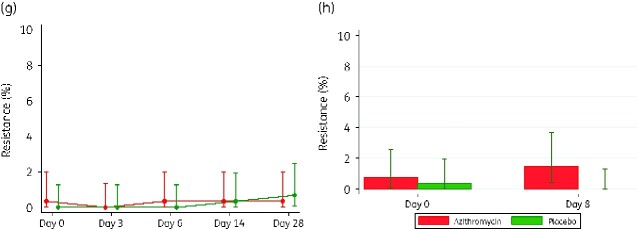
Continued.

**Table 3.** Prevalence of *K. pneumoniae* carriage and resistance by study arm

Characteristic	Azithromycin (%) (*n = *276)	Placebo (%) (*n = *288)	PR (95% CI)	*P* value
Prevalence of carriage				
Maternal NPS				
Pre^a^	3 (1.1)	2 (0.7)	1.57 (0.26–9.30)	0.619
Post^b^	12 (4.4)	16 (5.6)	0.78 (0.38–1.62)	0.509
Breast milk				
Post^b^	38 (13.8)	25 (8.7)	1.59 (0.98–2.56)	0.055
Vaginal swab				
Pre^a^	11 (4.0)	4 (1.4)	2.87 (0.92–8.90)	0.055
Post^c^	16 (5.8)	4 (1.4)	4.17 (1.41–12.33)	0.004^e^
Neonatal NPS				
Post^d^	41 (14.9)	31 (10.8)	1.38 (0.89–2.13)	0.145
Prevalence of carriage of resistant isolates				
Maternal NPS				
Pre^a^	0	0	0	0
Post^b^	0	1 (0.4)	0	1.000
Breast milk				
Post^b^	10 (3.6)	3 (1.0)	3.48 (0.97–12.51)	0.050
Vaginal swab				
Pre^a^	2 (0.7)	1 (0.4)	2.08 (0.19–22.88)	0.617
Post^c^	4 (1.5)	0	–	0.057
Neonatal NPS				
Post^d^	4 (1.5)	2 (0.7)	2.09 (0.39–11.30)	0.442

PR, prevalence ratio. *P* values from χ^2^ test for prevalence of carriage and Fisher’s exact test for prevalence of resistance.

a Incidence of carriage/resistance at day 0, pre-treatment.

b Cumulative incidence of carriage/resistance, i.e. proportion positive at one or more post-treatment visits (days 3, 6, 14 and 28).

c Incidence of carriage/resistance at day 8.

d Cumulative incidence of carriage/resistance, i.e. proportion positive at one or more post-treatment visits (days 0, 3, 6, 14 and 28).

e After adjusting for baseline imbalance, the difference was not significant (PR = 3.11, 95% CI 0.82–11.84, *P* = 0.095).

For newborns, there was a non-significant trend of higher prevalence in the NPS for *K. pneumoniae* in the azithromycin arm compared with the placebo arm (Table 3). Comparisons at 1 year were not possible because there was no *K. pneumoniae* isolated from infants’ NPS at this timepoint.

#### Prevalence of resistance

For study women, post-intervention prevalence of carriage of *K. pneumoniae* azithromycin resistant isolates was very low. It was similar for both arms in the nasopharynx, but higher in the azithromycin arm for both BM (3.6% versus 1.0% PR = 3.48 (0.97–12.51) *P = *0.050) and VS (1.5% versus 0% *P = *0.057). Details of prevalence of azithromycin-resistant isolates at each visit are shown in Figure 3(e-h). For newborns, nasopharyngeal carriage of *K. pneumoniae* azithromycin resistance was also low and similar in both arms (Table 3).

## Discussion

One oral dose (2 g) of intrapartum azithromycin did not reduce the carriage of the most common Gram-negative pathogens associated with neonatal sepsis, namely *E. coli* and *K. pneumoniae*, during the 4 weeks following the intervention; rather, *K. pneumoniae* prevalence increased significantly in BM and VS. In addition, prevalence of carriage of azithromycin-resistant *E. coli* and *K. pneumoniae* isolates increased, at least in the short term.

Although we had previously shown that intrapartum azithromycin significantly reduces the carriage of Gram-positive bacteria,^8^ the effect of the intervention on Gram-negative organisms had not been evaluated. The lack of effect on *E. coli* and *K. pneumoniae* carriage in all sample types is consistent with macrolides not being effective against most Enterobacterales, except for *Salmonella* and *Shigella* spp., owing to poor membrane permeability.^14^^,^^15^ A small study reported azithromycin to be effective against a pathogenic strain of *E. coli* when administered in combination with eculizumab and meningococcal vaccine in patients with haemolytic uraemic syndrome (HUS).^16^ Similarly, its effectiveness against *K. pneumoniae* is achieved only as adjunctive treatment.^17^

Data on the effect of azithromycin on the carriage of other Gram-negative bacteria, besides Enterobacterales, is also scarce. A small trial conducted in The Gambia showed that azithromycin 20 mg/kg administered on days 0, 7 and 14 had no effect on the oropharyngeal carriage of *Haemophilus influenzae.*^18^ A trial conducted in Texas, USA, showed a non-significant decrease in the nasopharyngeal carriage of *H. influenzae,* from 32% to 23%, in children with acute otitis media after azithromycin treatment.^19^ In our intrapartum azithromycin trial, we had shown a non-significant (probably because of low power) reduction of Gram-negative neonatal conjunctivitis.^20^

Our data also shows an increase of azithromycin resistance in *E. coli* and *K. pneumoniae* following the intervention, especially in VS. A possible explanation would be that although azithromycin reaches the vagina, it is ineffective for the reduction of carriage of these bacteria but still has the potential to select for resistance. A similar increase of resistance in the vaginal tract above the other biological sites was also observed in the main trial for *S. aureus* and Group A streptococci. *K. pneumoniae* resistance increased also in the BM, where we had previously shown a high concentration of azithromycin during the 4 weeks following the intervention.^21^ Such an increase in resistance had not been observed for previously studied Gram positives, in which we rather observed a major decrease in carriage without an increase in resistance. We did not observe any short-term increase of resistance in the Enterobacterales isolated from NPS from women in the study.

The prevalence of carriage of *K. pneumoniae* was not significantly different between arms in NPS from newborns, nor did we observe any increase in resistance from NPS isolated from newborns. We could not evaluate the long-term effects of the intervention on newborns because all NPS collected 12 months after the intervention were negative for *K. pneumoniae.*

There is a scarcity of data assessing the effect of prophylactic azithromycin on resistance in Gram-negative bacteria. In Tanzania, a follow-up study of a mass drug administration (MDA) of a single dose of oral azithromycin (20 mg/kg) in children aged between 6 months and 3 years evaluated antimicrobial resistance in *E. coli* isolated from rectal swabs. Resistance increased from 10% at baseline to 44% after the first month post-treatment, and then decreased to 23%, a figure still above the baseline levels.^22^ For Gram-positive bacteria, the effect on resistance seems to be dependent on the level of resistance at baseline,^23^ but data are lacking for a baseline resistance of <10% in Gram-negative bacteria. The effect on the gut could not be investigated in our study due to limitations of sample type. However, the possibility of sustained selective pressure with repeated administration as shown in another Tanzanian study 4 years following four rounds of annual azithromycin MDA, where *E. coli* resistance was at 16.6% (107/644), suggests a need for continuous monitoring.^23^

The study was designed to investigate the impact of the intervention on the main Gram-positive bacteria causing neonatal sepsis and was therefore underpowered to analyse its effect on Gram-negative bacteria as they have lower prevalence in the analysed samples. To increase such power, we based the analysis of Enterobacterales on a combined prevalence of carriage at any post-intervention timepoint during the follow-up as opposed to individual timepoints used previously for the Gram-positive bacteria.^8^ Other limitations were that we did not have rectal swabs or stools, the most suitable sample type, especially for *E. coli*, and it was not possible to evaluate how long the increased resistance initially observed lasted.

Although intra-partum azithromycin has been shown to decrease bacterial carriage of Gram-positive bacteria responsible for maternal and neonatal sepsis (*S. aureus*, *S. pneumoniae* and GBS),^8^^,^^9^ we have shown in this study that it does not reduce the carriage of Enterobacterales. The potential for inducing a short-term increase of azithromycin resistance among Enterobacterales underscores the importance of continuous monitoring of resistance in *E. coli* and *K. pneumoniae* as these bacteria are responsible for an important burden of resistance in hospital- and community-acquired infections. Future studies should further investigate the effect on resistance beyond the neonatal period and include rectal samples.
